# CRISPR/Cas9 Edited RAS & MEK Mutant Cells Acquire BRAF and MEK Inhibitor Resistance with MEK1 Q56P Restoring Sensitivity to MEK/BRAF Inhibitor Combo and KRAS G13D Gaining Sensitivity to Immunotherapy

**DOI:** 10.3390/cancers14215449

**Published:** 2022-11-05

**Authors:** Elizabeth Turner, Luping Chen, John G. Foulke, Zhizhan Gu, Fang Tian

**Affiliations:** R & D, American Type Culture Collection (ATCC), Gaithersburg, MD 20877, USA

**Keywords:** drug resistance, BRAF, KRAS, MEK, CRISPR/Cas9

## Abstract

**Simple Summary:**

BRAF inhibitor drug resistance has been a long-time challenge in the treatment of melanoma with *BRAF V600E* mutation. This study employed the CRISPR/Cas9 technology to generate three isogenic A375 melanoma cell lines with point mutations of *NRAS Q61K*, *KRAS G13D* and *MEK1 Q56P*, respectively. They recapitulated the resistance to BRAF inhibitors in vitro as such mutations have been found in patients with acquired resistance to BRAF inhibitors during treatment. Hence, these novel isogenic cell lines become extremely useful tools for upcoming research in this field. Additionally, we determined that resistance in the NRAS and MEK isogenic lines is driven by constitutive MEK/ERK signaling, while the resistance in the KRAS isogenic line is driven by EGFR overexpression. The *KRAS G13D* isogenic line displays elevated PD-L1 expression suggesting the *KRAS G13D* mutation could be a potential indication for immunotherapy.

**Abstract:**

*BRAF V600E* mutation drives uncontrolled cell growth in most melanomas. While *BRAF V600E* tumors are initially responsive to BRAF inhibitors, prolonged treatment results in inhibitor resistance and tumor regrowth. Clinical data have linked the *NRAS Q61K*, *KRAS G13D* and *MEK1 Q56P* mutations to the BRAF inhibitor resistance. However, development of novel therapeutics is hindered by the lack of relevant isogeneic cell models. We employed CRISPR/Cas9 genome engineering to introduce *NRAS Q61K*, *KRAS G13D* and *MEK1 Q56P* mutations into the A375 melanoma cell line with endogenously high expression of *BRAF V600E*. The resulting isogenic cell lines are resistant to BRAF inhibitors. The A375 *MEK1 Q56P* isogenic cells are additionally resistant to MEK inhibitors as single agent, but interestingly, these cells become sensitive to MEK/BRAF inhibitor combo. Our results suggest that resistance in the NRAS and MEK isogenic lines is driven by constitutive MEK/ERK signaling, while the resistance in the KRAS isogenic line is driven by EGFR overexpression. Interestingly, the *KRAS G13D* isogenic line displays elevated PD-L1 expression suggesting the *KRAS G13D* mutation could be a potential indication for immunotherapy. Overall, these three novel isogenic cell models with endogenous level *RAS* and *MEK1* point mutations provide direct bio-functional evidence demonstrating that acquiring a drug-resistant gene drives tumor cell survival and may simultaneously introduce new indications for combo therapy or immunotherapy in the clinic.

## 1. Introduction

More than half of all clinical melanomas carry an activating BRAF mutation, 90% of which are *BRAF V600E* [1,2,3]. The *BRAF V600E* oncogene functions by increasing signaling through the Ras/RAF/MEK/ERK pathway controlling cell survival and proliferation. Small molecule BRAF inhibitors such as dabrafenib and vemurafenib are highly effective for initial treatment of *BRAF V600E* melanomas. However, continued use of BRAF inhibitors leads to the accumulation of secondary activating mutations in Ras/RAF/MEK/ERK pathway genes that bypass BRAF inhibition, resulting in regrowth of BRAF-inhibitor resistant tumors [4,5,6,7,8]. Because of the genetic heterogeneity commonly observed in tumor samples, it is unclear if these secondary mutations were already present in some sub-populations of *BRAF V600E* tumor cells, or if BRAF inhibitor treatment itself drives the accumulation of additional mutations. Currently available cell-based models of drug-resistant melanoma are derived either from *BRAF V600E* melanomas that have been subjected to prolonged small molecule BRAF inhibition, or from cells overexpressing exogenous copies of the gene in question from a non-native promoter [9,10,11]. No study has yet demonstrated whether specific secondary mutations found in BRAF-inhibitor resistant tumor samples are the direct cause of BRAF inhibitor resistance, or if such genetic variants are only associated with resistance. Thus, the precise molecular and genetic mechanisms of acquired BRAF inhibitor resistance in *BRAF V600E* melanomas have yet to be defined, and this lack of mechanistic understanding hinders the development of novel chemotherapeutics and combination therapies effective for the treatment of tumors that have become resistant to BRAF inhibitor treatment regimens.

In this study, we use CRISPR/Cas9 gene editing to introduce three point mutations associated with acquired BRAF inhibitor resistance directly into A375 human malignant melanoma cells, which carry the *BRAF V600E* mutation. Use of CRISPR/Cas9 to directly engineer BRAF-inhibitor resistant melanoma cells provides a unique opportunity to study mutations associated with BRAF-inhibitor resistance in the absence of any selective drug pressure or exogenous protein expression and enable such mutant alleles to be expressed from the native promoter at physiologically relevant levels. Furthermore, because these mutations have been precisely engineered using CRISPR/Cas9 rather than by selective drug pressure, each new engineered cell line is genetically identical to the cell line from which it was derived, except for the introduced point mutation. Thus, each engineered model cell line can be used together with its parental line as an isogenic pair, and any observed difference in drug-resistance phenotype can be directly attributed to the introduced point mutation.

We find that the *KRAS G13D*, *NRAS Q61K*, and *MEK1 Q56P* mutations confer resistance to the BRAF inhibitors dabrafenib and vemurafenib in *BRAF V600E* A375 cells. The *MEK1 Q56P* mutation also renders the cells resistant to MEK inhibitors such as trametinib and binimetinib. Interestingly, *MEK1 Q56P* A375 cells are more sensitive to treatment with a combination of MEK and BRAF inhibitors than to either inhibitor in isolation, at equivalent doses. Additionally, the *NRAS Q61K* and *KRAS G13D* mutations bypass BRAF inhibition via distinct mechanisms: resistance in *KRAS G13D* cells appears to be primarily mediated by an overexpression of EGFR, while resistance in *NRAS Q61K* cells is caused by a failure of inhibition of downstream MEK-ERK signaling. Finally, PD-L1 expression is constitutively elevated in *KRAS G13D* but not in *NRAS Q61K* or *MEK1 Q56P* A375 cells, pointing to a possible role for immune checkpoint inhibition in melanomas carrying this specific biomarker.

These new CRISPR/Cas9 engineered isogenic models of acquired BRAF inhibitor resistance in *BRAF V600E* melanoma represent an important step forward for both the study of acquired BRAF inhibitor resistance and for the screening and development of novel chemotherapeutics and treatment regimens for patients with inhibitor resistant melanoma.

## 2. Materials and Methods

### 2.1. CRISPR/Cas9 Engineering of Ras and MEK1 Point Mutations

Point mutations were introduced into the NRAS, KRAS, and MEK1 genes by lipid-based co-transfection of two Cas9/gRNA all-in-one plasmid constructs targeting the introns to either side of the relevant coding exon (TransfeX, ATCC, ACS-4005). Cas9/gRNA all-in-one plasmids were derived from pcDNA3 and expressed tracrRNA and gRNA from a U6 promoter, and human codon optimized Cas9 and PuroR from a CMV promoter. Cells were also transfected with a pUC19-derived homologous sequence donor plasmid containing the desired point mutation and flanking exonic and intronic sequence. The following guide sequences were used: *NRAS Q61K* upstream intron 5′-TAGGGCCAGGTTATAATTGG**TGG**-3′ downstream intron 5′-ACATAAGTGAAGCTTGCTAC**TGG**-3′, *KRAS G13D* upstream intron 5′-GCATTTTTCTTAAGCGTCGA**TGG**-3′ downstream intron 5′-GTATTTCAGAGTTTCGTGAG**AGG**-3′, *MEK1 Q56P* upstream intron 5′-CATGTTGGTGATAGTCATCC**CGG**-3′ downstream intron 5′-AAAATTCAAACAGCACGGCT**GGG**-3′.

### 2.2. Cell Culture and Single Cell Cloning

The melanoma cell line A375 (ATCC, CRL-1619) and the derived isogenic lines A375 *KRAS G13D* (ATCC, CRL-1619IG-1), A375 *NRAS Q61K* (ATCC, CRL-1619IG-2) and A375 *MEK1 Q56P* (ATCC, CRL-1619IG-3) were grown and maintained in ATCC-formulated Dulbecco’s Modified Eagle’s Medium (ATCC, 30–2002) with 10% fetal bovine serum (ATCC, 30–2020). Cells were maintained at 37 °C and 5% CO_2_ in a humidified incubator.

For the establishment of isogenic cell lines, A375 cells were seeded at 1 × 10^5^ cells per well into 12-well culture plate and transfected with Cas9-sgRNA all-in-one plasmid and donor plasmids using TransfeX reagent. The transfected cell cultures were then enriched by puromycin selection. Single cells were isolated by automated cell sorting (Sony SH800) into 96-well plates and expanded for approximately 10–14 days. Once the single clones reached over 70% confluence per well, clones were then sub-cultured.

### 2.3. Genotyping for Positive KI Mutant Clones

Genomic DNA was extracted from CRISPR/Cas9 edited A375 clones using QuickExtract DNA extraction solution (Epicentre/Lucigen) according to manufacturer instructions. The CRISPR/Cas9 targeted genomic regions for each cell line were then PCR amplified and analyzed by Sanger sequencing for the presence of the desired point mutation. The following PCR primers were used: *NRAS Q61K* 5′- TGCATGTTTGTCTTCCTGAGA-3′ and 5′-ATCTTTTCCATTACATACTGAAAGATG-3′, *KRAS G13D* 5′-CCCCATGACACAATCCAGCTT-3′ and 5′-TCTGCTGCTGGTCTTTACTTTGG-3′, *MEK1 Q56P* 5′- GAGGATTTCTACTGTTGTGATTCAG-3′ and 5′-GGTATCCTGGCATTCCTTCCACAG-3′. The following nested sequencing primers were also used: *NRAS Q61K* 5′-GCAGGCATATAGAATTTGGTGGGTTTTCTTTTATGTAGGGTG-3′ and 5′- CTCTGGTTCCAAGTCATTCCCAGTAGCAAGC-3′, *KRAS G13D* 5′-AAGCGTCGATGGAGGAGTTTGTAAATGAAGTACAG-3′ and 5′-CATCATGGACCCTGACATACTCCCAAGGA-3′, *MEK1 Q56P 5′*-CAGACCTGGAGCTTTCTTTCCATGA-3′ and 5′-CTGGTCCCCAGGCTTCTAAGTAC-3′. Thermocycler parameters to amplify the 550 bp KRAS, 600 bp NRAS and 369 bp MEK1 on-target regions were as follows: step one, 98 °C for 1 min; step two, 98 °C 30 s; step three, 72 °C for 30 s, step four, 72 °C 30 s, repeat steps 2–4 for 29 times, step five, 72 °C for 4 min. PCR products were analyzed on 1.2% agarose double tier Lonza FlashGel. Positive KI mutant clones were identified by Sanger sequencing of PCR products.

### 2.4. Immunoblots

Cells were lysed in 1% SDS in phosphate buffered saline (PBS) followed by sonication for 3 × 5 s at 30% amplitude (QSonica CL-18). Lysates were then clarified by centrifugation at 13,000 RPM for 10 min in a table-top microcentrifuge and protein concentration was measured by bicinchoninic acid assay. Protein was loaded at 20 μg per lane on 4–12% gradient gels and migrated according to standard protocols. Protein was transferred to a nitrocellulose membrane using a quick dry blotting system (iBlot 2, ThermoFisher), which was then blocked with 5% nonfat milk protein in TBS-T. Blots were then probed with the following primary antibodies: rabbit monoclonal anti-EGF receptor (Cell Signaling Technology, 2232), rabbit monoclonal anti-p44/42 MAPK (ERK1/2) (Cell Signaling Technology, 4695), rabbit monoclonal anti-phospho-p44/42 (Thr202/Tyr294) (Cell Signaling Technology, 4370), rabbit monoclonal anti-MEK1/2 (Cell Signaling Technology, 9122), rabbit monoclonal anti-phospho-MEK1/2 (Ser217/221) (Cell Signaling Technology, 9121), rabbit monoclonal anti-AKT (Cell Signaling Technology, 4691), rabbit monoclonal anti-phospho-Akt (Ser473) (Cell Signaling Technology), rabbit monoclonal anti-PD-L1 (Abcam, ab213524), and rabbit polyclonal anti-GAPDH (Millipore-Sigma, G9545). Blots were then probed with goat anti-rabbit IgG (H + L)-HRP conjugate (Bio-Rad, 1706515) and bands were visualized using a Gel Doc XR+ gel documentation system (Bio-Rad).

### 2.5. D Cell Culture Dose-Response Curves

Cells were seeded in triplicate in 96-well culture plates at 2000 cells per well and grown overnight in the absence of any drug treatment. The following day, the indicated concentration of drug or an equivalent volume of DMSO vehicle control was added and the cells were grown for an additional three days. After three days of treatment, cell viability was assessed using CellTiter-Glo luminescent cell viability agent (Promega) according to manufacturer instructions. Luminescence was measured with a SpectraMax i3x multi-mode plate reader. The cell survival rate of drugged cells was normalized to the DMSO control group. The dose-response curves and IC50 values were generated using Prism (GraphPad). The BRAF inhibitors dabrafenib and vemurafenib, the MEK inhibitors trametinib and binimetinib, and the non-specific chemotherapeutic doxorubicin were obtained from ApexBio and Selleckchem.

### 2.6. D Spheroid Formation, Drug Treatment, and Imaging

Cells were seeded in 96-well ultra-low attachment microplates (Corning) at 500 cells per well and grown for 72 h. After 72 h the indicated concentration of drug was added, and the spheroids were grown for an additional 72 h. At the end of the 6-day time course spheroids were co-stained with NucBlue Live ReadyProbes reagent (Thermo Fisher, 37605) and 2.0 μM Calcein AM Green for two hours and then imaged by confocal microscopy on a Cellinsight CX7 high content analysis platform. For each spheroid, 15 confocal 20-micron Z-stacks were imaged in both the blue and green channels to visualize NucBlue-stained nuclei and Calcein AM Green-stained cytosol, respectively. The resulting 30 images were then assembled into a single image projection and analyzed using HCS Studio cell analysis software (Thermo Fisher). Total spheroid visible area was calculated by delineating the boundary of the spheroid in the green channel.

### 2.7. Flow Cytometry

A375 parental cells and A375 isogenic cells were harvested with non-enzymatic cell dissociation solution, washed once in ice cold PBS, and re-suspended in flow cytometry staining buffer at 1 × 10^6^ cells per mL. Then 200 μL of each cell type was stained with 20 μL R-phycoerythrin (PE) conjugated mouse anti-human CD274 (PD-L1) antibody (BD Pharmingen, 557924) for 30 min. Mouse IgG1k-PE (BD Pharmingen, 555749) was as an isotype control. Stained cells were analyzed by flow cytometry. Where indicated, cells were stimulated overnight with 200 ng/mL recombinant human interferon gamma (R & D Systems, 285-IF) before they were harvested for flow cytometry analysis.

## 3. Results

### 3.1. CRISPR/Cas9 Engineering of KRAS G13D, NRAS Q61K and MEK1 Q56P A375 Melanoma Models

**Figure 1:**
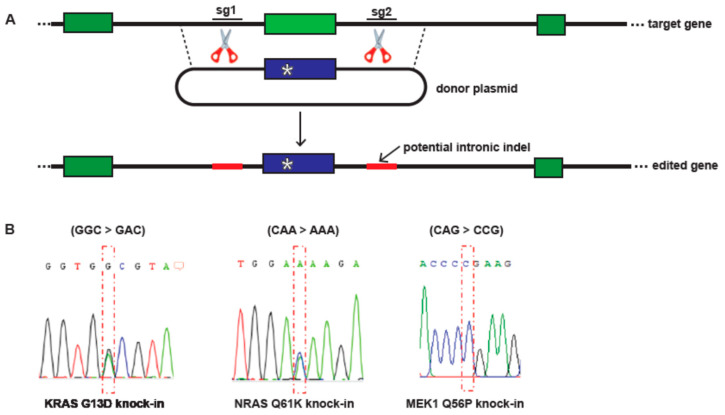
CRISPR/Cas9 engineering of isogenic A375 models of drug-resistant melanoma. (**A**) Schematic diagram of the CRISPR/Cas9 editing strategy used for the introduction of point mutations associated with BRAF and MEK inhibitor resistance into A375 melanoma cells. Two guide RNAs in complex with Cas9 (sg1 and sg2, scissors) were used to create double-stranded breaks in the intronic regions (black lines) to either side of the target exon (black box). A donor plasmid containing a copy of the target exon with the desired point mutation (star) and flanking intronic sequences was used as a repair template. This strategy ensures that any indels resulting from imperfect sequence repair at the Cas9 cut sites (red lines) are spliced out during mRNA processing and do not affect the resulting cellular protein; (**B**) Sanger sequencing of genomic DNA from the resulting KRAS G13D heterozygous (left), NRAS Q61K heterozygous (middle), and MEK1 Q56P homozygous (right) A375 isogenic lines.

To generate inhibitor resistant model cell lines, three specific point mutations associated with BRAF inhibitor resistance in clinical samples were introduced into *BRAF V600E* A375 cells using CRISPR/Cas9 genome engineering. The three mutations, *NRAS Q61K*, *KRAS G13D*, and *MEK1 Q56P*, were selected based on the strength of the clinical data supporting their association with BRAF inhibitor resistance, and because all three mutations affect the Ras/RAF/MEK/ERK signaling pathway [10,11]. CRISPR/Cas9 gene editing can be error-prone, resulting in random insertions and deletions at the guide RNA target site if the double-stranded break made by Cas9 is repaired via non-homologous end-joining. We therefore used a gene editing strategy in which two guide RNAs were used to cut the genomic DNA in the introns to either side of the target exon, then the entire exon and flanking intronic sequence was replaced, via homologous recombination, with a donor sequence carrying the desired point mutation (Figure 1A). This method both increases the frequency of homology-directed repair over non-homologous end joining [12] and ensures that any random insertions or deletions caused by error-prone cellular DNA repair mechanisms are spliced out during mRNA processing and do not affect the coding sequence of the resulting protein. All three engineered melanoma models in this study were derived by homology-directed repair, with no alteration to the intronic sequence.

After CRISPR/Cas9 gene editing, edited pools were sorted into single cells, which were then expanded and sequenced for the presence of the desired point mutation (Figure 1B). Single-cell clones carrying heterozygous *KRAS G13D* c.41 G > A, heterozygous *NRAS Q61K* c.184 C > A, and homozygous *MEK1 Q56P* c.170 A > G point mutations were obtained from this screen. Total cellular RNA was then harvested from each candidate clone and the cDNA sequences of expressed *KRAS*, *NRAS*, and *MEK1* genes, respectively, were obtained (Supplementary Figure S1). In each case, the coding sequence of the relevant gene carried the desired point mutation and was free from any random insertions or deletions resulting from the introduction of double-stranded DNA breaks.

Finally, candidate clones were screened for the presence of random insertions and deletions at selected off-target cutting sites, and for Cas9/gRNA plasmid integration. Off-target sites for screening were selected based on their location in a known coding sequence, the number of mismatches from the on-target sequence, and the predicted *in silico* off-target cutting score [13]. Integration of the plasmid CMV promoter, but not of the Cas9 coding sequence, was detected in A375 *KRAS G13D* and A375 *NRAS Q61K*, and no plasmid integration was detected in *MEK1 Q56P* (Supplementary Figure S6). Six off-target cut sites for each of the two guide RNA constructs used in the generation of A375 *KRAS G13D* were tested, and no off-target cutting was detected (Supplementary Figure S4). Similarly, no off-target cutting was detected at any of the six off-target sites selected for the two guides used in the construction of A375 *NRAS Q61K* (Supplementary Figure S3), or in either of the ten off-target sites corresponding to the guides used in the construction of *MEK1 Q56P* (Supplementary Figure S5). These results, together with the use of guide RNAs selected based on predicted in silico cutting efficiency and target sequence specificity, rather than on proximity to the desired point mutation, indicate that the resulting mutant A375 cell lines differ from parental A375 cells only with respect to the desired point mutation. Each mutant cell line can thus be tested alongside parental A375 cells as an isogenic pair, and any observed differences in cellular phenotype, such as BRAF inhibitor resistance can, with high confidence, be attributed directly to the introduced point mutation.

### 3.2. RAS and MEK Mutant A375 Melanoma Models Are Resistant to BRAF and MEK Inhibitors in 2D Tissue Culture

**Figure 2:**
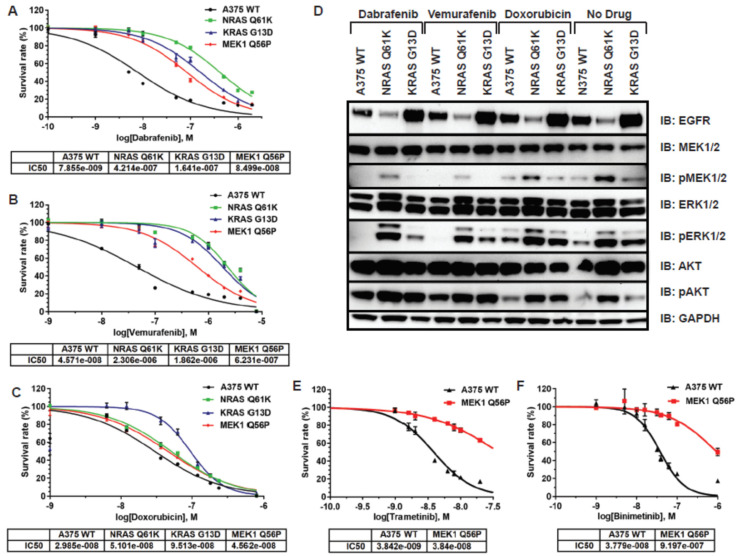
Isogenic melanoma models are resistant to BRAF inhibitors but not to BRAF non-specific chemotherapeutics in 2D tissue culture. (**A**) Dabrafenib resistance of A375 melanoma models in 2D tissue culture; (**B**) Vemurafenib resistance of A375 melanoma models in 2D tissue culture; (**C**) No resistance to the BRAF non-specific chemotherapeutic doxorubicin in 2D tissue culture; (**D**) Immunoblot demonstrating BRAF inhibitor resistance in EGFR pathway signaling in NRAS Q61K and KRAS G13D A375 melanoma models. Cells were treated with 1.0 μM of the indicated drug for 90 min prior to harvesting protein; (**E**) Dose-response curve for the MEK inhibitor trametinib in the MEK1 Q56P melanoma model in 2D tissue culture; (**F**) The same curve as in (**E**) for the MEK inhibitor binimetinib.

Because *NRAS Q61K*, *KRAS G13D*, and *MEK1 Q56P* mutations are associated with BRAF inhibitor resistance in *BRAF V600E* melanoma, we first tested each A375 model cell line for resistance to the FDA-approved BRAF inhibitor compounds dabrafenib and vemurafenib in 2D tissue culture. Each cell line was subjected to increasing concentrations of BRAF inhibitor, then cell viability was determined for each drug condition and IC50 values were calculated from the resulting dose-response curves (Figure 2A,B). All three mutant cell lines were found to be resistant to both BRAF inhibitors compared to control A375 cells. The IC50 of unmodified A375 cells to dabrafenib was determined to be on the order of 10^−9^, while the introduction of RAS (*NRAS Q61K* and *KRAS G13D*) and *MEK1 Q56P* mutations yielded dabrafenib resistance on the order of 10^−7^ and 10^−8^ representing a 100- and 10-fold decrease in sensitivity, respectively. A similar trend in vemurafenib resistance was obtained with A375, RAS and *MEK1 Q56P* mutation cell lines yielding IC50 values in the order of 10^−8^, 10^−6^ and 10^−7^, respectively. In order to determine whether the observed BRAF inhibitor resistance phenotypes were specific to BRAF inhibition, rather than to a general insensitivity to small molecule compounds, all four cell lines were tested for resistance to the BRAF non-specific chemotherapeutic doxorubicin. Doxorubicin is a DNA intercalator that induces apoptosis selectively in rapidly dividing cell populations and should thus be unaffected by the presence of engineered RAS and MEK point mutations. As expected, no significant difference in doxorubicin sensitivity was detected between WT and RAS or MEK mutant A375 cells, with all four lines having doxorubicin IC50 values on the order of 10^−8^ (Figure 2C).

BRAF inhibitors such as dabrafenib and vemurafenib inhibit cell proliferation and survival by decreasing the serine/threonine-protein kinase activity of BRAF in the Ras/RAF/MEK/ERK signaling pathway that drives both normal and aberrant cell division [14,15]. Resistance to BRAF inhibitors in *BRAF V600E* melanoma develops when secondary mutations and/or changes in basal protein expression allow for the activation of MEK/ERK signaling even in the presence of BRAF inhibitors [16,17,18,19]. To further understand the impact of *NRAS Q61K* and *KRAS G13D* mutations on Ras/RAF/MEK/ERK pathway signaling in our *BRAF V600E* melanoma models, western blotting was carried out for the A375 WT, *NRAS Q61K*, and *KRAS G13D* cells after treatment with either dabrafenib, vemurafenib, doxorubicin, or vehicle control, then total cellular protein was harvested for immunoblotting. Lysates were probed with antibodies against EGFR, MEK1/2, phospho-MEK1/2 (Ser217/221), ERK1/2, phospho-ERK1/2 (Thr202/Tyr204), AKT, and phosphor-AKT (Ser473) (Figure 2D). Basal total EGFR expression was reduced relative to WT A375 cells in the *NRAS Q61K* line, and significantly elevated relative to WT in the *KRAS G13D* line in all conditions, a result that is consistent with reports of resistance to anti-EGFR therapy in colorectal cancer patients with confirmed *KRAS G13D* mutation [20]. No changes in MEK1/2 expression were detected in either cell line regardless of drug treatment, but phosphorylation of MEK1/2 was dramatically reduced after treatment with BRAF inhibitor in WT and *KRAS G13D*, but not in *NRAS Q61K* A375 cells, indicating a differential impact on MEK signaling between the two RAS mutant melanoma models. A similar pattern of perturbation was observed for ERK1/2, which operates directly downstream of MEK1/2 in this signaling pathway. Phosphorylation of ERK was completely inhibited after both dabrafenib and vemurafenib treatment in WT A375 cells, significantly inhibited in *KRAS G13D* A375 cells, but not inhibited in *NRAS Q61K* A375 cells, which maintained high levels of both basal ERK expression and phosphorylation of ERK regardless of inhibitor treatment. Interestingly, phosphorylation of AKT, which operates in parallel to MEK and ERK in MAP kinase signaling, was increased relative to WT in both *NRAS Q61K* and *KRAS G13D* A375 cells, both in the presence and absence of inhibitor. These results indicate that while *KRAS G13D* mutation drives BRAF inhibitor resistance primarily through an increase in basal EGFR expression, *NRAS Q61K* mutation decreases the extent to which downstream MEK and ERK signaling is abrogated by upstream RAF inhibition.

Finally, because MEK1 mutation is associated with both BRAF and MEK inhibitor resistance in *BRAF V600E* melanoma [18], we also tested the *MEK1 Q56P* A375 melanoma model for resistance to the FDA-approved MEK inhibitor compounds trametinib and binimetinib. *MEK1 Q56P* A375 cells were found to have a ~10-fold lower sensitivity to trametinib and a ~30-fold lower sensitivity to binimetinib relative to unmodified A375 cells (Figure 2E,F).

### 3.3. Ras and MEK1 Mutant Melanoma Models Display Differential Ras/RAF/MEK/ERK Pathway Perturbations in 2D vs. 3D Tissue Culture

Three-dimensional tissue culture is an important tool for pre-clinical drug development because it allows for the evaluation of drug resistance phenotypes in a format that more closely mimics the human tumor microenvironment. A375 cells readily form 3D spheroids when grown in an ultra-low attachment spheroid microplate. WT A375, A375 *NRAS Q61K*, *KRAS G13D*, and *MEK1 Q56P* cells were able to reproducibly form spheroids in 3D tissue culture.

**Figure 3:**
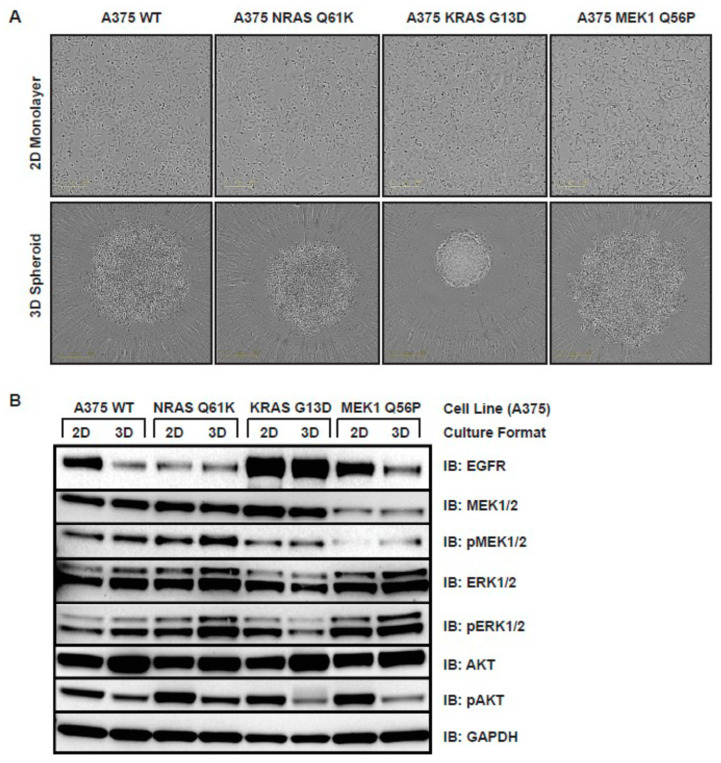
Effect of tissue culture format on EGFR pathway signaling in drug-resistant melanoma models. (**A**) A375 WT, NRAS Q61K, KRAS G13D, and MEK1 Q56P cells growing in a 2D monolayer (top) and as 3D spheroids (bottom). For spheroid formation, 500 cells from each line were seeded in each well of a 96-well ultra-low attachment spheroid microplate and grown for five days before imaging; (**B**) Immunoblots tracking EGFR pathway signaling in engineered A375 melanoma model cells in 2D and 3D tissue culture. Protein was harvested from each cell line growing in 2D culture, and cellular protein from 3D spheroids was collected from spheroids seeded at 500 cells per 96-well and grown for seven days. Total protein (20 μg) was loaded in each lane and samples were blotted for total EGFR, total MEK1/2, phospho-MEK1/2 (Ser217/221), total ERK1/2 (p44/42 MAPK), phospho-ERK1/2 (Thr202/Tyr204), AKT, phospho-AKT (Ser473), and GAPDH.

In order to assess any differences in Ras/RAF/MEK/ERK signaling between A375 melanoma models in 2D versus 3D tissue culture, WT, *NRAS Q61K*, *KRAS G13D*, and *MEK1 Q56P* A375 cells were grown in either 2D or 3D tissue culture then total cellular protein was harvested for immunoblotting. As before, lysates were probed with antibodies against EGFR, MEK1/2, phospho-MEK1/2, ERK1/2, phospho-ERK1/2, AKT, and phospho-AKT (Figure 3B). Basal EGFR expression was found to be constitutively elevated in A375 *KRAS G13D* relative to A375 WT regardless of culture format, and constitutively lowered in *NRAS Q61K* relative to WT regardless of culture format. However, for both A375 WT and A375 *MEK1 Q56P*, EGFR expression was lower in 3D than in 2D tissue culture, indicating that activating RAS mutations control EGFR expression via a mechanism that is unaffected by the aggregate rate of cell division. Levels of both MEK1/2 and phospho-MEK1/2 were found to be lower relative to WT A375 cells in *MEK1 Q56P*, but not in either of the RAS mutant lines in both tissue culture formats. Furthermore, basal AKT expression was higher in 3D than in 2D tissue culture, but vice-versa for phospho-AKT in all four cell lines. This result is consistent with the lower average level of cell division in the hypoxic central core of a mature spheroid relative to the same cells grown in a 2D monolayer [21,22,23].

### 3.4. RAS and MEK1 Mutant A375 Isogenic Melanoma Models Are Resistant to BRAF Inhibitors in 3D Tissue Culture

Following characterization of the drug resistance phenotypes and MEK/ERK/MAPK signaling perturbations in A375 RAS and MEK1 mutant melanoma models in 2D tissue culture, we next evaluated the drug responses of the parental cell line and the three isogenic cell lines in a 3D culture environment. WT, *NRAS Q61K*, *KRAS G13D*, and *MEK1 Q56P* A375 spheroids were subjected to a three-day treatment with the BRAF inhibitors dabrafenib and vemurafenib, as well as the non-specific chemotherapeutic agent doxorubicin. Additionally, *MEK1 Q56P* spheroids were independently treated with the MEK inhibitors trametinib and binimetinib. Subsequently, spheroids were stained with live-cell nuclear stain and Calcein AM green (a fluorescein-derived metabolic marker that stains only live cells). Spheroid sizes were analyzed on a high-content imaging platform.

**Figure 4:**
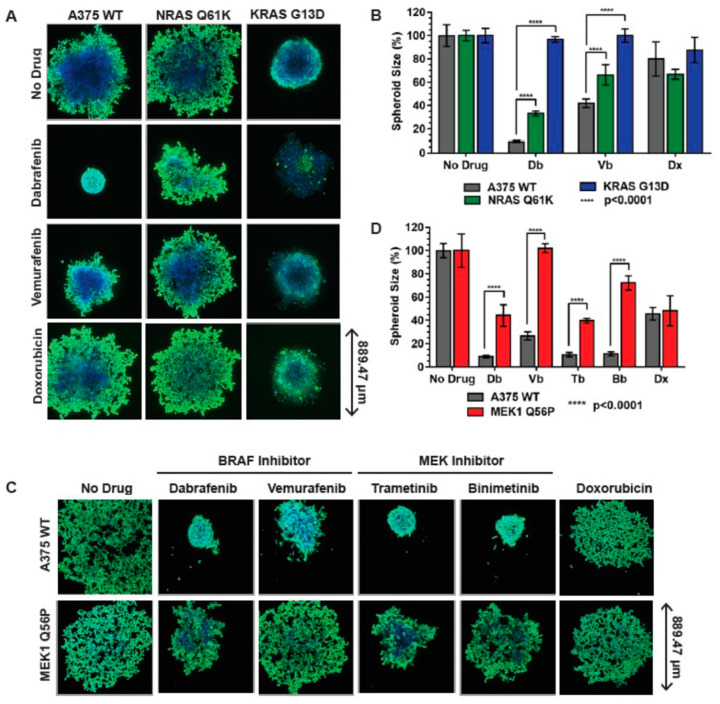
A375 Ras mutant melanoma models are resistant to BRAF inhibitors in 3D tissue culture and A375 MEK1 melanoma model is resistant to both MEK and BRAF inhibitors in 3D tissue culture. (**A**) BRAF inhibitor resistance in A375 RAS mutant melanoma models in 3D tissue culture. For each indicated cell type, 500 cells were seeded in each well of a ULA spheroid microplate and grown for three days in the absence of drug. After 72 h the indicated BRAF-specific inhibitor or the BRAF-nonspecific chemotherapeutic agent doxorubicin was added (Db = dabrafenib 25 nM, Vb = vemurafenib 50 nM, Dx = doxorubicin 100 nM) and the spheroids were grown for an additional three days. Spheroids were then stained with 2 μM Calcein AM green and NucBlue live-cell nuclear marker for 90 min and then imaged; (**B**) Average spheroid size of RAS mutant melanoma models following drug treatment relative to the un-drugged condition. Statistical analysis was performed using two-way ANOVA with multiple comparisons, each condition represents at least *n* = 3 spheroids; (**C**) MEK and BRAF inhibitor resistance in A375 MEKQ56P spheroid melanoma model. Drugged spheroids were handled as in (**A**) and treated with 25 nM dabrafenib, 50 nM vemurafenib, and 50 nM of the MEK inhibitors trametinib (Tb) and binimetinib (Bb), respectively, or 100 nM doxorubicin; (**D**) Average numbers of nuclei per spheroid were calculated relative to the un-drugged condition. Results from at least *n* = 3 spheroids for each condition were averaged and plotted, statistical analysis was performed using two-way ANOVA with multiple comparisons. ****, *p* < 0.0001.

As was observed in 2D tissue culture, *NRAS Q61K, KRAS G13D,* and *MEK1 Q56P* cells were significantly more resistant to the BRAF inhibitors dabrafenib and vemurafenib than the WT A375 cells from which they were derived (Figure 4A,C). Similarly, no resistance to the non-specific chemotherapeutic doxorubicin was observed for the RAS and MEK mutant spheroids. *MEK1 Q56P* A375 spheroids, like *MEK1 Q56P* cells grown in 2D tissue culture, were also more resistant to the MEK inhibitors trametinib and binimetinib than WT A375 cells.

Interestingly, all four A375 melanoma model lines were more sensitive to the MEK and BRAF-specific inhibitors in 3D tissue culture than in 2D tissue culture, requiring lower concentrations of inhibitor to achieve a reduction in spheroid size comparable to the loss of cell viability observed in 2D tissue culture. For example, A375 WT spheroid size was reduced to ~10% of the size of an untreated control A375 WT spheroid after three days of treatment with 25 nM dabrafenib (Figure 4A,B), but A375 WT viability was reduced to a similar level of viability (~20% of the untreated control condition) by three days of treatment with a much higher dose of 100 nM dabrafenib in 2D tissue culture (Figure S2A). Similarly, *NRAS Q61K* spheroids subjected to treatment with 50 nM vemurafenib were reduced to ~60% of the size of an untreated *NRAS Q61K* spheroid (Figure 4A,B), while treatment of *NRAS Q61K* cells in 2D tissue culture with up to 1 μM vemurafenib for the same amount of time yielded less than a 10% decrease in cell viability (Figure S2B). Conversely, all four A375 melanoma models are less sensitive to the non-specific chemotherapeutic doxorubicin in 3D tissue culture than they are in 2D tissue culture. This is presumably because, as a DNA intercalator, doxorubicin selectively affects rapidly dividing cell populations, and cells grown in 3D tissue culture form a non-replicating, senescent core with only the outer layers of cells maintaining active cell division.

These findings, in addition to demonstrating the resistance of engineered melanoma model cell lines to MEK and BRAF inhibitors in a 3D tissue culture system, clearly highlight the value of the 3D tissue culture format for the screening and optimization of novel chemotherapeutics and combination drug therapies for the treatment of drug-resistant melanomas.

### 3.5. MEK1 Q56P A375 Cells Are Sensitive to Combination BRAF/MEK Inhibitor Treatment in 2D and 3D Tissue Culture

**Figure 5:**
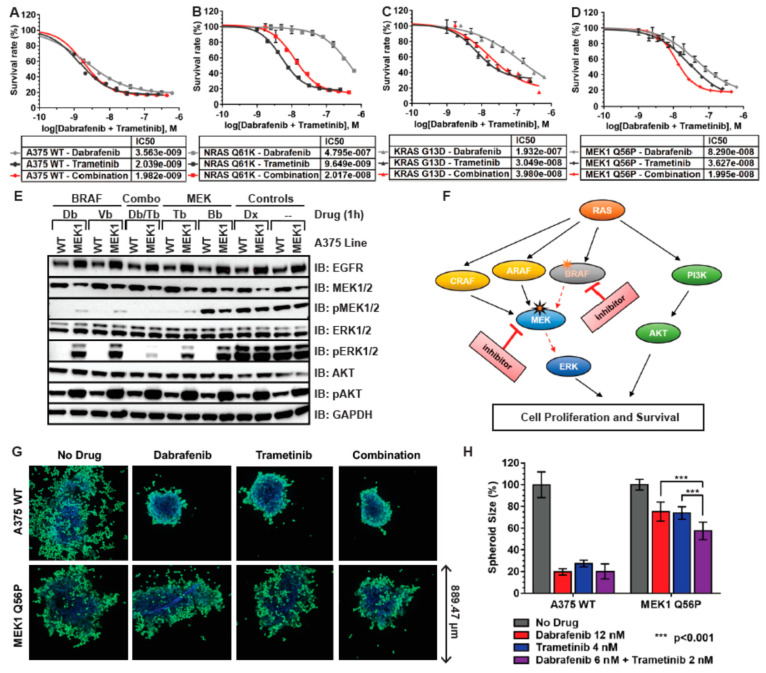
MEK1 Q56P melanoma model is sensitive to combination BRAF/MEK inhibitor treatment in both 2D and 3D tissue culture. (**A**) Dose-response curves for A375 WT cells in 2D tissue culture with dabrafenib (grey line), trametinib (black line), or a combination of dabrafenib and trametinib (red line, molarity indicates total drug concentration); (**B**) Dose-response curves for NRAS Q61K cells in 2D tissue culture with dabrafenib, trametinib, or a combination of dabrafenib and trametinib; (**C**) Dose-response curves for MEK1 Q56P cells in 2D tissue culture with dabrafenib, trametinib, or combination; (**D**) Dose-response curves for MEK1 Q56P cells in 2D tissue culture with dabrafenib, trametinib, or combination. Lower survival with combination indicates synergistic drug killing in this line; (**E**) Immunoblot demonstrating synergistic inhibition of the MEK/ERK signaling pathway in MEK1 Q56P melanoma model cells in 2D tissue culture. Cells were treated with either 1.0 μM of the indicated inhibitor compound or with 0.5 μM each of each indicated drug for 90 min prior to harvesting protein (Db = dabrafenib 1.0 μM, Vb = vemurafenib 1.0 μM, Tb = trametinib 1.0 μM, Bb = binimetinib 1.0 μM, Db/Tb = dabrafenib 0.5 μM + trametinib 0.5 μM); (**F**) Model of synergistic inhibition of the RAS/RAF/MEK/ERK pathway by combination MEK and BRAF inhibitor treatment. Orange star indicates the primary BRAF V600E mutation which drives cell proliferation in the absence of BRAF inhibitor. Treatment with BRAF inhibitor results in secondary MEK1 Q65P mutation (black outlined orange star). In the presence of these two mutations, upstream pathway inhibition with BRAF inhibitor in combination with downstream pathway inhibition with MEK inhibitor (red arrows) leads to less cell survival and proliferation than is observed when each drug is used alone; (**G**) Susceptibility of MEK1 Q56P cells grown in 3D tissue culture to dabrafenib, trametinib, and combination drug treatment. For each indicated cell type, 500 cells were seeded in each well of a ULA spheroid microplate and grown for three days in the absence of drug. The spheroids were then treated with 12 nM dabrafenib, 4 nM trametinib, a combination of 6 nM dabrafenib and 2 nM trametinib, or vehicle control for an additional three days. Spheroids were then stained with 2 μM Calcein AM green and NucBlue live-cell nuclear marker for 90 min and then imaged; (**H**) Spheroid sizes were calculated relative to the un-drugged condition. Results from at least *n* = 3 spheroids for each condition were averaged and plotted, statistical analysis was performed using two-way ANOVA with multiple comparisons. ***, *p* < 0.001.

After assessing the MEK and BRAF inhibitor resistance phenotypes of A375 melanoma model cell lines in both 2D and 3D tissue culture, we next evaluated each line for sensitivity to combination MEK/BRAF inhibitor treatment. Each cell line was treated in 2D tissue culture with either the BRAF inhibitor dabrafenib, the MEK inhibitor trametinib, or an equivalent combination drug treatment consisting of a half-dose of dabrafenib and a half-dose of trametinib (Figure 5A–C). A375 WT cells were found to have a similar level of sensitivity to MEK, BRAF, and combination inhibitor treatment in 2D tissue culture (Figure 4A), while RAS and MEK mutant lines were more sensitive to trametinib than to dabrafenib at all drug concentrations tested (Figure 4B–D). However, while the sensitivity of *NRAS Q61K* and *KRAS G13D* cells to combination drug treatment was intermediate to the sensitivity to each drug in isolation (Figure 5B,C), *MEK1 Q56P* cells were more sensitive to a half-dose of dabrafenib in combination with a half-dose of trametinib than to a full dose of either drug alone, across a wide range of drug concentrations (Figure 5D).

In order to examine the specific perturbations in Ras/RAF/MEK/ERK signaling underlying the sensitivity of *MEK1 Q56P* melanoma cells to combination MEK/BRAF inhibitor treatment, *MEK1 Q56P* cells were treated with either dabrafenib, vemurafenib, trametinib, binimetinib, doxorubicin, or a combination drug treatment consisting of a half-dose of dabrafenib plus a half-dose of trametinib. Total cellular protein was then analyzed by immunoblot for the basal levels of EGFR, MEK1/2, ERK1/2, and AKT expression, as well as the level of MEK1/2, ERK1/2 and AKT phosphorylation, which correlated with an increase in signaling through the Ras/RAF/MEK/ERK signaling pathway. As expected, *MEK1 Q56P* cells maintained phosphorylation of both MEK and ERK in the presence of BRAF and MEK inhibitors, while WT A375 cells had diminished MEK or ERK phosphorylation in the presence of these compounds (Figure 5E). This result demonstrates that *MEK1 Q56P* cells are resistant to MEK and BRAF inhibitor treatment because, unlike WT A375 cells, *MEK1 Q56P* cells maintain signaling for cell survival and proliferation via phosphorylation of MEK and ERK proteins even in the presence of inhibitors that completely abrogate MEK/ERK signaling in unmodified A375 cells. However, when *MEK1 Q56P* cells were treated with an equivalent dose of dabrafenib and trametinib in combination, phosphorylation of MEK and ERK decreased dramatically, to levels of Ras/RAF/MEK/ERK pathway signaling that more closely resemble the response of WT A375 cells to BRAF or MEK inhibitor treatment alone (Figure 5E, Db/Tb Combo condition).

Because A375 *MEK1 Q56P* melanoma cells carry the activating *BRAF V600E* mutation as well as an activating MEK1 mutation, inhibition of BRAF alone fails to abrogate aberrant proliferation signaling through downstream MEK proteins, while inhibition of MEK alone cannot effectively compensate for the increase in upstream BRAF signaling. Instead, by inhibiting Ras/RAF/MEK/ERK signaling at multiple points along the signaling cascade (Figure 5F), synergistic inhibition of cell proliferation can be achieved in a way that requires lower equivalent doses of each drug compound. Conversely, inhibition of MEK and BRAF in combination has very little impact on Ras/RAF/MEK/ERK signaling in RAS mutant A375 melanoma cells, as activating RAS mutations operate upstream of both MEK and BRAF in this signaling pathway.

Finally, we evaluated the response of *MEK1 Q56P* melanoma cells grown in 3D tissue culture to combination MEK/BRAF inhibitor treatment. Spheroids were treated for three days with either 12 nM dabrafenib, 4 nM trametinib, 6 nM dabrafenib together with 2 nM trametinib, or vehicle control, then spheroids were then stained and imaged (Figure 5G). As was observed in 2D tissue culture, A375 WT cells responded similarly to treatment with BRAF inhibitor, MEK inhibitor, and to an equivalent combination of MEK and BRAF inhibitor. However, *MEK1 Q56P* spheroids were more sensitive to the combination drug treatment condition than to an equivalent dose of dabrafenib or trametinib in isolation (Figure 5G,H). These results demonstrate that the engineered A375 *MEK1 Q56P* model of acquired BRAF inhibitor resistance, when used together with the isogenic WT A375 cells from which they were derived, is a valuable new tool for the screening and development of new combination therapies in both 2D and 3D tissue culture.

### 3.6. Elevated Basal PD-L1 Expression in A375 KRAS G13D Isogenic Melanoma Model

**Figure 6:**
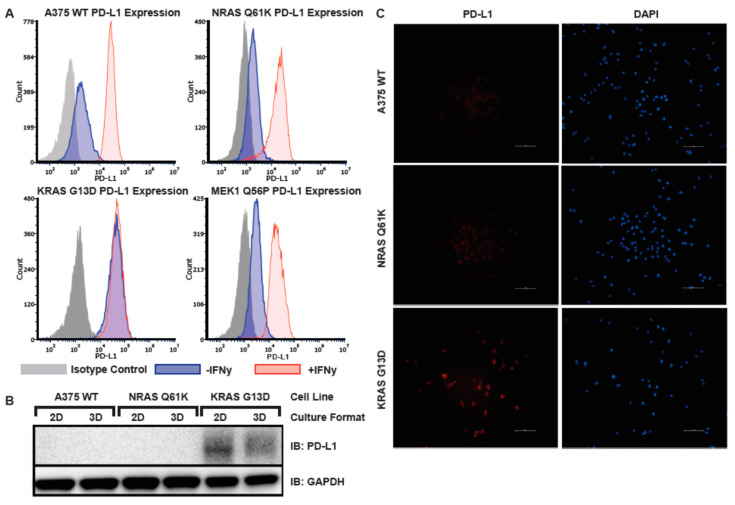
PD-L1 is constitutively expressed in KRAS G13D, but not in A375 WT, NRAS Q61K, or MEK1 Q56P melanoma models. (**A**) Flow cytometry analysis of cell surface PD-L1 expression in A375 WT, NRAS Q61K, KRAS G13D, and MEK1 Q56P melanoma models grown in 2D tissue culture. Cells were treated overnight with 200 ng/µL interferon gamma (red), or mock treated (blue). The following day the cells were stained with either anti-PD-L1 or isotype control (grey); (**B**) PD-L1 immunoblot of total cellular protein from A375 WT, NRAS Q61K, and KRAS G13D cells grown in either 2D or 3D tissue culture; (**C**) Indirect immunofluorescence staining of PD-L1 in A375 WT, NRAS Q61K and KRAS G13D melanoma model lines.

Because BRAF inhibitor resistance in *BRAF V600E* melanoma is associated with an immunosuppressed tumor microenvironment and consequent tumor immune evasion [24] we tested all three isogenic melanoma models for increased levels of cell-surface PD-L1 expression by flow cytometry (Figure 6A). We found elevated levels of PD-L1 expression in *KRAS G13D*, but not in *NRAS Q61K* or *MEK1 Q56P* A375 cells relative to WT. Furthermore, pre-treatment of cells with interferon gamma, which typically increases the cell surface expression of PD-L1, had no effect on PD-L1 expression in *KRAS G13D* cells, but increased PD-L1 expression as expected in WT, *NRAS Q61K*, and *MEK1 Q56P* A375 cells. This finding indicates that PD-L1 expression in A375 *KRAS G13D* is constitutively elevated in a way that is unaffected by immune signaling, and suggests that combination therapies involving anti-PD-L1 immune blockade inhibition together with inhibitors targeting the Ras/RAF/MEK/ERK signaling pathway could be effective for the treatment of a subset of BRAF-inhibitor resistant melanomas.

Next, in order to determine if the observed phenotype of constitutive cell-surface expression of PD-L1 in A375 *KRAS G13D* is due to an upregulation of trafficking of PD-L1 to the cell surface or to a general increase of cellular expression of PD-L1 protein, lysates from WT, *NRAS Q61K*, and *KRAS G13D* A375 cells grown in both 2D and 3D tissue culture were probed with anti-PD-L1 antibody (Figure 6B), and it was determined that total cellular PD-L1 expression was indeed elevated relative to WT in A375 *KRAS G13D* cells, and that the level of PD-L1 expression was unaffected by tissue culture format.

## 4. Discussion

In this study, we have demonstrated how CRISPR/Cas9 genome engineering can be used to create novel, physiologically relevant cell-based models of acquired BRAF inhibitor resistance in *BRAF V600E* melanoma. By using precision gene editing rather than selective drug pressure or exogenous overexpression of mutant alleles to generate drug-resistant model cell lines, we have conclusively demonstrated that three specific mutations associated with acquired BRAF-inhibitor resistance, *NRAS Q61K*, *KRAS G13D*, and *MEK1 Q56P*, are the direct cause of the observed drug-resistance phenotype. We have also confirmed that the molecular mechanism underlying BRAF-inhibitor resistance in these three melanoma models is the aberrant re-activation of Ras/RAF/MEK/ERK signaling, which is consistent with clinical data [25]. However, each isogenic model with secondary mutation has a distinct profile of aberrant Ras/RAF/MEK/ERK pathway activation. For example, while both *NRAS Q61K* and *KRAS G13D* drive MEK/ERK signaling in the presence of BRAF inhibitor, phosphorylation of MEK/ERK is much greater in *NRAS Q61K* than in *KRAS G13D* A375 cells after BRAF inhibitor treatment. However, both mutations confer a comparable degree of BRAF inhibitor resistance, suggesting that reactivation of MEK/ERK signaling predominantly accounts for the BRAF inhibitor resistance of *NRAS Q61K* cells, while it is likely that factors other than persistent MEK/ERK phosphorylation contribute to BRAF inhibitor resistance in *KRAS G13D* cells. Notably, we observe a significant increase in the basal levels of both EGFR and PD-L1 expression in *KRAS G13D* A375 melanoma cells but not in *NRAS Q61K* cells. This finding is consistent with patient data demonstrating that resistance to BRAF inhibitors in patients with secondary *KRAS G13D* mutations leads to an increase in basal EGFR expression by negative feedback through the Ras/RAF/MEK/ERK signaling pathway [26,27]. The observation of constitutive PD-L1 expression in the *KRAS G13D* but not in the *NRAS Q61K* melanoma model is further consistent with findings of elevated PD-L1 levels in tumors with activating KRAS mutations and with studies demonstrating elevated PD-L1 expression in non-small-cell lung cancers with activating EGFR mutations and/or overexpression of EGFR [28,29,30,31,32].

The resistance of our *MEK1 Q56P* melanoma model to both MEK and BRAF inhibitors confirms the prior discovery of BRAF inhibitor cross-resistance with stable expression of exogenous MEK1 Q56 and MEK1 P124 mutant alleles in A375 cells [18]. However, unlike other MEK1 mutations associated with resistance to MEK inhibitors, the *MEK1 Q56P* mutation falls outside allosteric drug binding pocket [33], indicating that this mutation confers resistance to MEK inhibitor by increasing the intrinsic kinase activity of MEK1 [34] or by an as-yet unknown mechanism. The suggested therapeutic benefit from our data depicting diminished combined MEK/BRAF inhibitor resistance in *MEK1 Q56P* A375 cells, but not in the *KRAS G13D* or *NRAS Q61K* melanoma models is consistent with clinical data demonstrating the effectiveness of combined MEK/BRAF inhibitor therapy for the treatment of melanomas with secondary activating RAS mutations and acquired BRAF inhibitor resistance [5].

The increased sensitivity of the A375 melanoma model cell lines to BRAF inhibitor in 3D relative to 2D tissue culture, and the increased sensitivity of the *MEK1 Q56P* isogenic A375 melanoma model to both MEK inhibitor and combination MEK/BRAF inhibitor when grown in 3D tissue culture highlight the utility of the 3D tissue culture format for high-throughput drug screening. The fact that all three melanoma models are also less sensitive to non-specific compounds such as doxorubicin in 3D than in 2D tissue culture further supports the idea that 3D tissue culture models are the more effective tool for the screening of targeted drugs than 2D cultures, and have the potential to significantly lower costs associated with expensive animal model. Furthermore, our results also show that there are significant differences in Ras/RAF/MEK/ERK pathway activation when the cells are grown in a 2D rather than a 3D culture format. Our finding of diminished basal AKT signaling in all four A375 melanoma models indicates that cells in 3D culture environment favor signaling for cell proliferation over signaling for cell survival in aggregate. It may also suggest that the 3D tissue culture format is particularly useful for screening inhibitors that affect the Ras/RAF/MEK/ERK signaling pathway.

Acquired BRAF inhibitor resistance presents a significant clinical challenge as there are only few effective treatment options for melanomas that have developed secondary BRAF-inhibitor resistance. In this study, we have shown how CRISPR/Cas9 genome engineering can be used for the generation of novel cell-based models of acquired inhibitor resistance, which are ideally suited for the screening of new inhibitor compounds and combination therapies targeting treatment-resistant melanomas. Given the rapidly evolving combination treatment landscape, future studies using the *KRAS G13D*/*BRAF V600E* isogenic model of drug-resistant melanoma described here should focus on combination PD-L1 and BRAF/MEK inhibitor treatment. Initial studies could be performed in a standard 2D tissue culture format, but further refinement of such a treatment regimen can be accomplished via 3D tissue culture, or even 3D co-culture of A375 melanoma model cells with stromal cells, which would more closely mimic the physiological tumor micro-environment. With high expression levels of PDL-1 and EGFR on the cell surface plus both *BRAF V600E* and *KRAS G13D* existing within its genome, the A375 *KRAS G13D* mutant isogenic line is an ideal model suited for future studies focused on the tumor microenvironment and integrated combination therapy strategies.

## 5. Conclusions

Melanoma remains the most lethal form of skin cancer exhibiting high mortality rates, due to a high likelihood of developing metastases and acquiring drug resistance. We used the CRISPR genome editing technology to target endogenous loci and knock in drug resistant mutation within the A375 melanoma cell line. The created isogenic cell lines represent a new type of drug resistance models that contains defined genetic resistance mechanisms, which provides an invaluable tool for developing next generation therapeutics that can overcome drug resistance in melanoma. Interestingly, *KRAS G13D* knock-in has dramatically increased both EGFR and PD-L1 expression levels, while isogenic cells carrying *NRAS Q61K* and *MEK Q56P* have constant activation of the MEK-ERK pathway. This indicates the expression of PD-L1 is directly linked to KRAS mutation or the downstream effects thereof, rather than to a general increase in Ras-Raf-MAPK signaling. It also suggests a potential utility of combining the targeted therapy with immunotherapy for clinical patients with acquired drug resistance.

## Data Availability

The data presented in this study are available on request from the corresponding author.

## Supplementary Materials

The following supporting information can be downloaded at: https://www.mdpi.com/article/10.3390/cancers14215449/s1, Figure S1: CRISPR/Cas9 Target-site cDNA sequencing of A375 melanoma models; Figure S2: Drug resistance and growth rates of A375 melanoma models in 2D tissue culture; Figure S3: Genomic loci screened for off-target Cas9 cutting in A375 NRAS Q61K; Figure S4: Genomic loci screened for off-target Cas9 cutting in A375 KRAS G13D; Figure S5: Genomic loci screened for off-target Cas9 cutting in A375 MEK1 Q56P; Figure S6: Cas9/CMV plasmid sequence integration screening of A375 NRAS Q61K, KRAS G13D, and MEK1 Q56P melanoma models.
