# Supplementary material for: CRISPR/Cas9 Edited RAS & MEK Mutant Cells Acquire BRAF and MEK Inhibitor Resistance with MEK1 Q56P Restoring Sensitivity to MEK/BRAF Inhibitor Combo and KRAS G13D Gaining Sensitivity to Immunotherapy
Source: Cancers (Basel). 2022 Nov 5;14(21):5449. doi: 10.3390/cancers14215449 (PMC9655013; doi:10.3390/cancers14215449)
Supplement: Supplementary file 1 [file cancers-14-05449-s001.zip › cancers-1964987-supplementary.pdf]

Supplementary Data

CRISPR/Cas9 edited RAS & MEK mutant cells acquire BRAF and MEK inhibitor resistance with MEK1 Q56P restoring sensitivity to MEK/BRAF inhibitor combo and KRAS G13D gaining sensitivity to immunotherapy

Elizabeth Turner[1], Luping Chen, John Foulke, Zhizhan Gu*, and Fang Tian*

[1] R&D, American Type Culture Collection (ATCC), Gaithersburg, MD, USA.
* Correspondence: zgu@atcc.org & ftian@atcc.org

Supplementary Figure Legends

Supplementary Figure S1. CRISPR/Cas9 Target-site cDNA sequencing of A375 melanoma models. A. Exon structure of unprocessed (top) and processed (bottom) NRAS mRNA. Red arrows indicate the location of primers used for PCR amplification and Sanger sequencing of NRAS cDNA, green arrow indicates location of the translation start codon **B.** Sanger sequencing chromatogram of NRAS Q61K knock-in target site cDNA expressing heterozygous NRAS c.184 C>A point mutation. **C.** Exon structure of unprocessed (top) and processed (bottom) KRAS mRNA. Red arrows indicate the location of primers used for PCR amplification and Sanger sequencing of KRAS cDNA, green arrow indicates location of the translation start codon. **D.** Sanger sequencing chromatogram of KRAS G13D knock-in target site cDNA expressing heterozygous KRAS c.41 G>A point mutation. **E.** Exon structure of unprocessed (top) and processed (bottom) MEK1 mRNA. Red arrows indicate the location of primers used for PCR amplification and Sanger sequencing of MEK1 cDNA, green arrow indicates location of the translation start codon. **F.** Sanger sequencing chromatogram of MEK1 Q56P knock-in target site cDNA expressing homozygous MEK1 c.170 A>G point mutation. G. Sequence of primer pairs used for amplification and Sanger sequencing of NRAS, KRAS, and MEK1 CRISPR/Cas9 target sites.

Supplementary Figure S2. Drug resistance and growth rates of A375 melanoma models in 2D tissue culture. A. Survival rates by CellTiter-Glo viability assay of A375 WT, NRAS Q61K, KRAS G13D, and MEK1 Q56P melanoma models at 1 nM, 10 nM, and 100 nM dabrafenib. Statistical analysis was performed by two-way ANOVA with multiple comparisons, n=3 for each condition. **B.** Cell survival rates as in (A) at 10 nm, 100 nm, and 1 µM vemurafenib. **C.** Survival rates by CellTiter-Glo viability assay of A375 WT and MEK1 Q56P melanoma models at 1 nM, 4 nM, and 10 nM trametinib. Statistical analysis was performed by two-way ANOVA with multiple comparisons, n=3 for each condition. **D.** Cell survival rates as in (C) at 10 nM, 100 nM, and 1 µM binimetinib. **E.** Growth rates of A375 WT, NRAS Q61K, KRAS G13D, and MEK1 Q56P melanoma models in 2D tissue culture, as measured by confluence.

Supplementary Figure S3. Genomic loci screened for off-target Cas9 cutting in A375 NRAS Q61K. A. Off-target cutting screen for NRAS knock-in sgRNA1 with off-target sequence, genomic

coordinates, number of mismatches from the target sequence, and sequence of primers used for amplification and Sanger sequencing of each off-target locus. Off-target sites were selected based on number of mismatches relative to the target sequence and by proximity to known coding regions. No indels were detected. **B.** The same as in (A) for NRAS knock-in sgRNA2. No indels were detected.

Supplementary Figure S4. Genomic loci screened for off-target Cas9 cutting in A375 KRAS G13D. A. Off-target cutting screen for KRAS knock-in sgRNA1 with off-target sequence, genomic coordinates, number of mismatches from the target sequence, and sequence of primers used for amplification and Sanger sequencing of each off-target locus. Off-target sites were selected based on number of mismatches relative to the target sequence and by proximity to known coding regions. No indels were detected. **B.** The same as in (A) for KRAS knock-in sgRNA2. No indels were detected.

Supplementary Figure S5. Genomic loci screened for off-target Cas9 cutting in A375 MEK1 Q56P. A. Off-target cutting screen for MEK1 knock-in sgRNA1 with off-target sequence, genomic coordinates, number of mismatches from the target sequence, and sequence of primers used for amplification and Sanger sequencing of each off-target locus. Off-target sites were selected based on number of mismatches relative to the target sequence. No indels were detected. B. The same as in (A) for MEK1 knock-in sgRNA2. No indels were detected.

Supplementary Figure S6. Cas9/CMV plasmid sequence integration screening of A375 NRAS Q61K, KRAS G13D, and MEK1 Q56P melanoma models. A. Cas9/CMV plasmid sequence integration screening of A375 NRAS Q61K. **B.** Cas9/CMV plasmid sequence integration screening of A375 KRAS G13D. **C.** Cas9/CMV plasmid sequence integration screening of A375 MEK1 QQ56P. **D.** Primer pairs used in Cas9/CMV plasmid integration screening.

Supplementary Figure S1

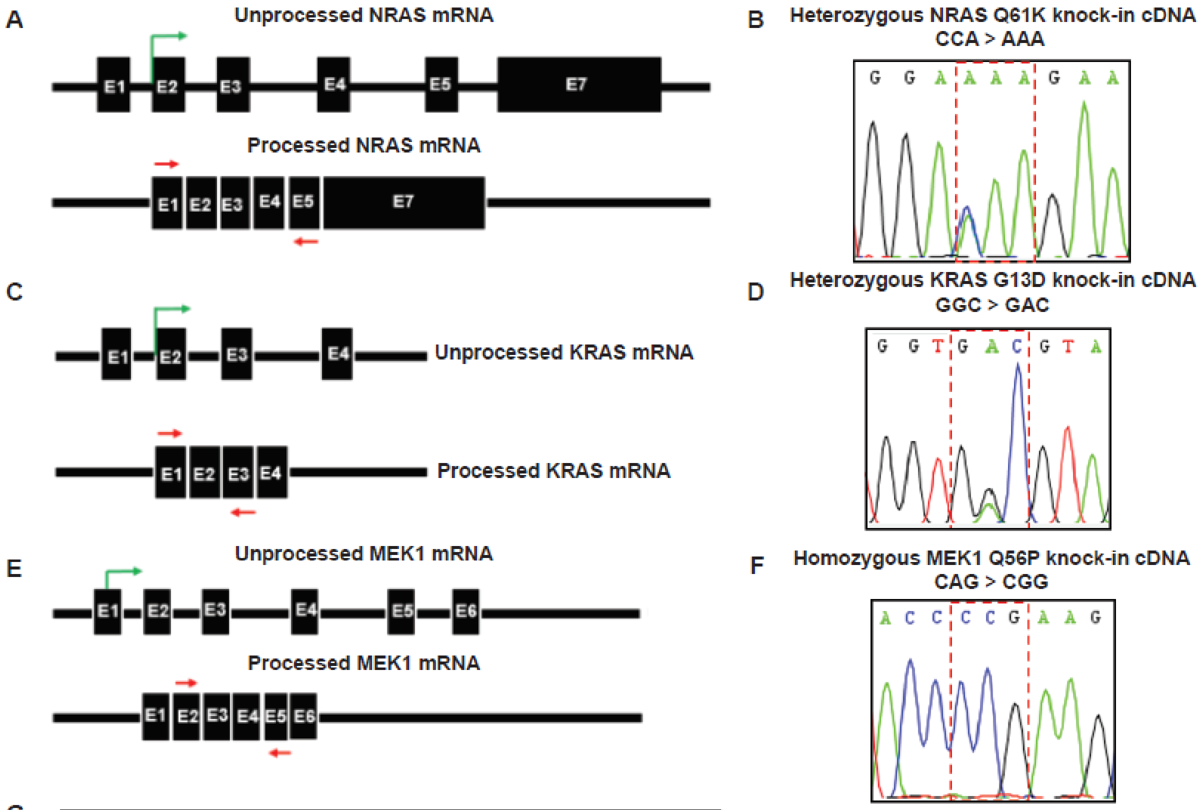

G

Primer Name	Primer Sequence (5' to 3')	Amplicon
NRAS-cDNA-FW	GGCTGTGGTCCTAAATCTGTC	594 bp
NRAS-cDNA-RV	CCCATCATCACTGCTGTTGAG	
KRAS-cDNA-FW	CCGCCATTTCGGACTGGGAGCGA	457 bp
KRAS-cDNA-RV	TCACATTTATTTCCTACTAGGACCATAGG	
MEK1-cDNA-FW	CTTGGAGGCCTTGCAGAAGAAGCTGG	493 bp
MEK1-cDNA-RV	GATCATGCACAGAGATGTCAAGCCC	

Supplementary Figure S2

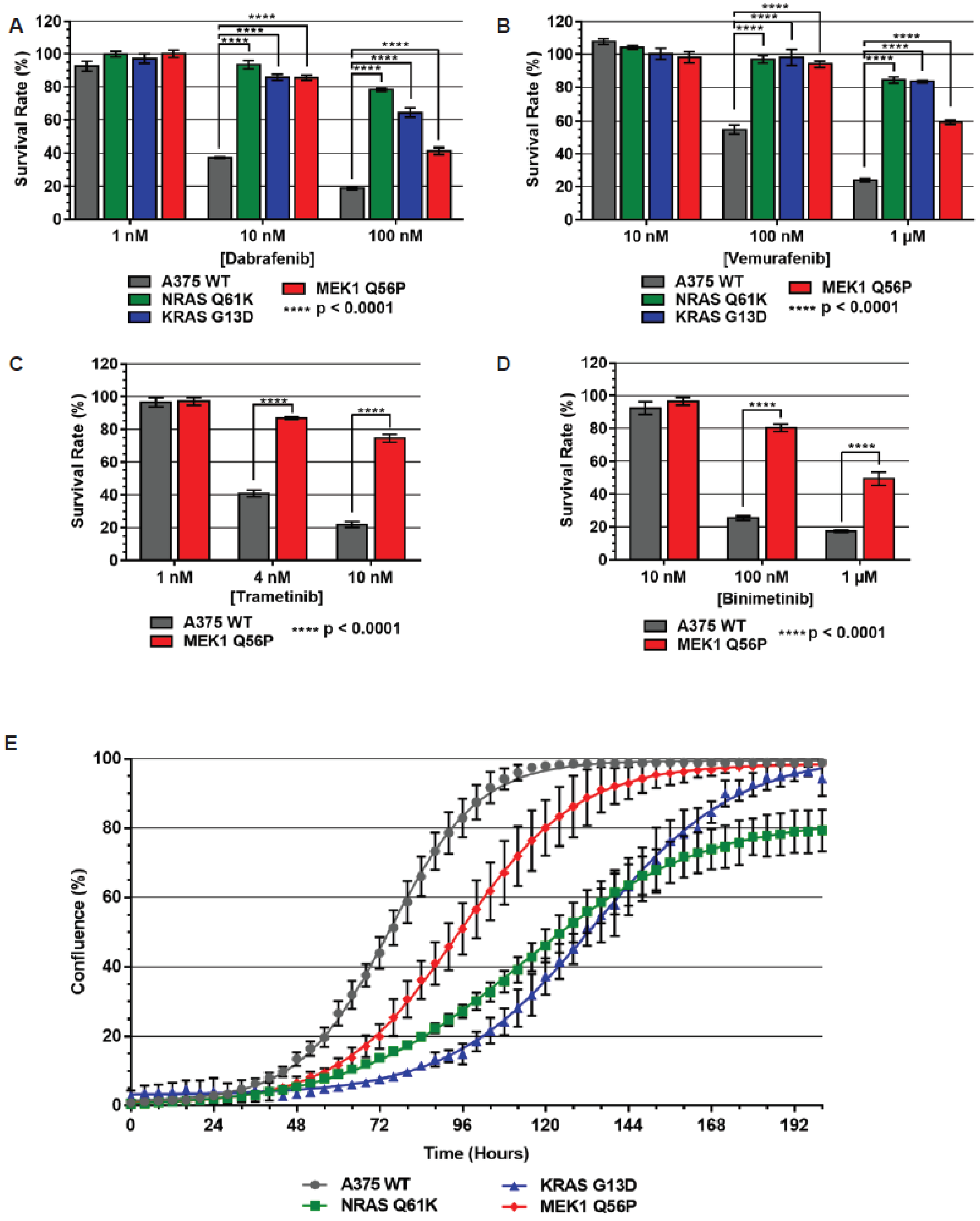

Supplementary Figure S3

A

Target	Coordinates	Strand	MM	Target Sequence	PAM	Gene	Primer Sequences (5' to 3')	Result
NRAS sgRNA1 Target	chr1:114714381-114714403	-	0	TAGGGCCA[GGTTATAATTGG]	TGG	NRAS	TGCATGTTTGTCTTCCTGAGA<br>ATCTTTTCCATTACATACTGAAAGATG	N/A
NRAS sgRNA1 OT1	chr19:39083209-39083231	+	3	TACGGGGA[GGTTATAATTGG]	GGG	PAPL	CCCAACACCTTGTATCACAATAGC<br>CACATAGGCTTCTACTGATTGTGG	No Indel
NRAS sgRNA1 OT2	chr13:74391887-74391909	-	3	TAGTGTCA[GATTATAATTGG]	AGG	RPL21P108	GGTGTGGACATTATAAAGCTAAACATGTAA<br>CCAGGCACAGAGTACTTGTTACGGAAGTGT	No Indel
NRAS sgRNA1 OT3	chrX:107907663-107907685	-	3	GAGGGACA[GGTGATAATTGG]	AGG	MID2	CAGAGAGCAGACATTGAGAATGAGGAGA<br>GGAAGAGATGTCTCTTCATTTGTGCTTTGG	No Indel
NRAS sgRNA1 OT4	chr1:176489495-176489517	+	4	TGGTGACA[CGTTATAATTGG]	TAG	PAPPA2	GGATTTTAATGCATAGAGGAGCTTCC<br>GAGATTCAGCATCCTGCTTGTCCATA	No Indel
NRAS sgRNA1 OT5	chr2:232785583-232785605	+	4	TTAGTCCA[GCTTATAATTGG]	GGG	GIGYF2	GGGTTTGATCCACTTCCAGACTCAC<br>GAGATTCAGCATCCTGCTTGTCCATA	No Indel
NRAS sgRNA1 OT6	chr2:195608816-195608838	+	4	TGCAGCCA[GGCTATAATTGG]	CAG	SLC39A10	GTGTCATGAGGGCTAAGTGCAGTAA<br>CCAGCCGTGGGAGGTTATTTCAGAC	No Indel

B

Target	Coordinates	Strand	MM	Target Sequence	PAM	Gene	Primer Sequences (5' to 3')	Result
NRAS sgRNA2 Target	chr1:114713566-114713588	-	0	ACATAAGT[GAAGCTTGCTAC]	TGG	NRAS	TGCATGTTTGTCTTCCTGAGA<br>ATCTTTTCCATTACATACTGAAAGATG	N/A
NRAS sgRNA2 OT1	chr5:141428584-141428606	-	3	TCTTCAGT[GAAGCTTGCTAC]	CAG	PCDHGB8P	GCCCACACAGAAAGGAACAGGAAAT<br>GGTTGCTTTCACCCTGGTCTCTGT	No Indel
NRAS sgRNA2 OT2	chr6:6363617-6363639	-	3	AAACAAGT[GCAGCTTGCTAC]	CAG	LY86-AS1	GCAAAGCCTGTTGACAAAAGTCCTTG<br>GCCTGAGAATACTCTTGTCTCTCATCTT	No Indel
NRAS sgRNA2 OT3	chr3:60951631-60951653	+	3	AAAGAAGT[GCAGCTTGCTAC]	CAG	FHIT	ACAATAAGGCAGCCCATGACCAA<br>CCCATCTAGATCATTCTCCTTAAATACT	No Indel
NRAS sgRNA2 OT4	chr4:186040203-186040225	+	3	ACAGTAGT[TAAGCTTGCTAC]	CAG	RNU4-64P	GTTCTGTGCAAACGCCGAGCCATTGAT<br>CCCATATAAGCTCTGGAGTATTAAGGAC	No Indel
NRAS sgRNA2 OT5	chr4:24464902-24464924	-	4	GCAGAGGG[GAAGCTTGCTAC]	TGG	AC092846.1	GGATTCCTTGAGTCCAGGCGTTCAAGAC<br>CCCATATAAGCTCTGGAGTATTAAGGAC	No Indel
NRAS sgRNA2 OT6	chr2:113212262-113212284	+	4	CTGTCAGT[GAAGCTTGCTAC]	CAG	PAX8-AS1	GCAGTTAAGTCTTCTGGGTGCTCTCA<br>CTCCAGTTTCAGGAGCCCGAGCTCCT	No Indel

Supplementary Figure S4

A

Target	Coordinates	Strand	MM	Target Sequence	PAM	Gene	Primer Sequences (5' to 3')	Result
KRAS sgRNA1 Target	chr12:25245605-25245627	-	0	GCATTTTT[CTTAAGCGTCGA]	TGG	KRAS	CCCCATGACACAATCCAGCTT<br>CTGCTGCTGGTCTTTACTTTGG	N/A
KRAS sgRNA1 OT1	chr11:119058359-119058381	+	3	GCATTTTC[CTTAAGTGTGGA]	TGG	HYOU1	GTGCCTGTAATCCCAGCTACTCACGA<br>GACCACTCTCCACCTAAGCATCACGAAA	No Indel
KRAS sgRNA1 OT2	chr8:108920528-108920550	+	3	GCATCTTT[CTTAAGCATTGA]	GGG	RP11-1084E5.1	GCTCCAAGATCACCGTCTTCTCTCTAC<br>GTCACTTCATGCTATGAGAAGTTCTGCA	No Indel
KRAS sgRNA1 OT3	chr9:5939470-5939492	+	4	GAATCTTT[CTTAATCATCGA]	TGG	KIAA2026	GTTATCATTCCTTACAATATCTTCAGTTA<br>ATCACTGAAATCATAATACCAGATCCCATC	No Indel
KRAS sgRNA1 OT4	chrX:120553913-120553935	+	4	ACATTCTT[TTTAAGCGTAGA]	GGG	CUL4B	GCAACATAGCAAGACCCCCATATCTC<br>GAGCTCTGAAGCCACACGATTTTAAATTC	No Indel
KRAS sgRNA1 OT5	chr16:87963594-87963616	+	4	GGACTTTT[CTTAAGGGTTGA]	TGG	BANP	GACTGGCCGCTCTGCATGGATCTGAT<br>GTCAAGCCAGGCTTCCTGTATTTAACACT	No Indel
KRAS sgRNA1 OT6	chr15:60162524-60162546	-	4	GCATAATT[CTTAAGCATGGA]	AGG	RP11-82L7.4	GCTGCAGTTGGATTGGATCGGACTT<br>GTGCAGAGTGCCAGCAAGCTGGTTT	No Indel

B

Target	Coordinates	Strand	MM	Target Sequence	PAM	Gene	Primer Sequences (5' to 3')	Result
KRAS sgRNA2 Target	chr12:25245015-25245037	-	0	GTATTTCA[GAGTTTCGTGAG]	AGG	KRAS	CCCCATGACACAATCCAGCTT<br>CTGCTGCTGGTCTTTACTTTGG	N/A
KRAS sgRNA2 OT1	chr18:73343401-73343423	+	4	GCTTTTCA[GACGTTCGTGAG]	AGG	CTD-2354A18.1	GCCAAGCTGGTCTTGACCTCCTGA<br>CCTCGTACCTCACTGATCACTTTTGCC	No Indel
KRAS sgRNA2 OT2	chr5:126961339-126961361	+	4	AAATTTCA[GAGAATCGTGAG]	AGG	3-Mar	GCAAGCACAAACACAGGTCCTTCCTAC<br>GCATAACTGTTGGCATTTATTCCTGCCCTT	No Indel
KRAS sgRNA2 OT3	chr3:102032478-102032500	+	4	GAACTTCT[GAGTTTAGTGAG]	TGG	RP11-221J22.2	TGTGAAGTGCTTACTGAACATCTTTGTGG<br>CCCACAATGTTATTTTGCTCGAGGC	No Indel
KRAS sgRNA2 OT4	chr15:41054090-41054112	-	4	GTTTTGAA[GAGTTTGGTGAG]	AGG	INO80	CAACCCTATTAGTGATAAAATGTCCCTAA<br>GAGGTCTTCTTTACTTACCTTGTCAGA	No Indel
KRAS sgRNA2 OT5	chr6:144838615-144838637	-	4	GTGTTATA[GAGTTTTGTGAG]	GGG	UTRN	CAGCTCATACTCCAAGGCAATATTGAGA<br>GTAACATCTGCATTCCCTATATGATGG	No Indel
KRAS sgRNA2 OT6	chr16:3756542-3756564	-	4	CCATTTCA[GGGTTTTGTGAG]	TGG	CREBBP	GACTTGCATGAGTGCTGCTTCCTA<br>GGCACATGGACACTTACACGCTG	No Indel

Supplementary Figure S5

A

Target	Coordinates	Strand	MM	Target Sequence	PAM	Gene	Primer Sequences (5' to 3')	Result
MEK1 sgRNA1 Target	chr15:66434832-66434854	+	0	CATGTTGG[TGATAGTCATCC]	CGG	MEK1	GAGGATTTCTACTGTTGTGATTCAG<br>GGTATCCTGGCATTCCTTCCACAG	N/A
MEK1 sgRNA1 OT1	chr1:10853864-10853886	-	3	AATGATGG[TGACAGTCATCC]	TGG	HSPE1P24	ACCACCACCACAACCATTGCTAC<br>TGGTGATGTGGATGTTGATCAGGATAGT	No Indel
MEK1 sgRNA1 OT2	chr22:29816431-29816453	+	3	CAAGTTGG[AGTTAGTCATCC]	AGG	ASCC2	CAAATGGTTGCCAGCAGGTCTGTG<br>ATGCTCGAACCTACGCACTCAC	No Indel
MEK1 sgRNA1 OT3	chr2:86435176-86435198	-	3	CATGTTTT[TGAGAGTCATCC]	AGG	KDM3A	CTGAGGGAGGAGAATTGGTTG<br>TCTATGGCTGATTTCACACTACAG	No Indel
MEK1 sgRNA1 OT4	chr20:4745349-4745371	-	3	AATGTTGG[TGTCAGTCATCC]	TGG	PRNT	GCGTGATCCCTTTGCCCCATTGT<br>GGAGCCATAAGCTCTCACGTCTGT	No Indel
MEK1 sgRNA1 OT5	chr11:108532803-108532825	-	4	CAAGTATG[AGATAGTCATCC]	AGG	EXPH5	CTCTTGAAGCAAGGCTAGCCA<br>CTTGCATTCCCAGGCTGTACTC	No Indel
MEK1 sgRNA1 OT6	chr19:5663907-5663929	+	4	ACTCTTGG[TGAAAGTCATCC]	TGG	SAFB	CGAGAGAGATGGCAGGTGCACTT<br>GGTCCCTGTGGTCAAAGTCGTGG	No Indel
MEK1 sgRNA1 OT7	chr3:185058989-185059011	-	4	CTTTTTGA[TCATAGTCATCC]	TGG	VPS8	GTGGGCAGATCACCTGAGTCTAG<br>GCCACGTTGTGGGATTGCAGGAT	No Indel
MEK1 sgRNA1 OT8	chr8:137426391-137426413	-	4	GATAATGG[TGACAGTCATCC]	AGG	ZYXP1	GCATGGTGGCAGGCGACTATAA<br>GCCTGTGGAAAGTGAAGTCGGAAA	No Indel
MEK1 sgRNA1 OT9	chr15:29974505-29974527	-	4	CATTTTCT[TAATAGTCATCC]	CGG	TJP1	GGGCAACACAGTGGGACTTCATC<br>CCTTGCTTTGAGTTTATTCTGCTCCTCTT	No Indel
MEK1 sgRNA1 OT10	chr3:112605446-112605468	+	4	CATGATGA[CGGTAGTCATCC]	TGG	CCDC80	GAGGTTTCAATAATAACTCATGAATAGG<br>CCCAATGTGGTCCATGGTGATTGTG	No Indel

B

Target	Coordinates	Strand	MM	Target Sequence	PAM	Gene	Primer Sequences (5' to 3')	Result
MEK1 sgRNA2 Target	chr15:66435621-66435643	-	0	AAAATTCA[AACAGCACGGCT]	GGG	MEK1	GAGGATTTCTACTGTTGTGATTCAG<br>GGTATCCTGGCATTCCTTCCACAG	N/A
MEK1 sgRNA2 OT1	chr9:128918779-128918801	-	4	AAAAATTA[GCCAGCACGGCT]	GGG	LRRC8A	AATGCAGTGGCTCGATCTC<br>CCTAGCTTCATGCACATCCT	No Indel
MEK1 sgRNA2 OT2	chr10:126245050-126245072	+	3	CAAATGCA[AACAGCAAGGCT]	GGG	RP11-81B10.2	GGGACAGGAATGGGGGACTAGAAA<br>GTGCCCTTTCCAAAGCACAGACTG	No Indel
MEK1 sgRNA2 OT3	chr18:51488559-51488581	-	3	AAAAATTA[AACAGCATGGCT]	GGG	RP11-267C16.1	CTCCCAAGTAGCTGAGACTAAAG<br>CGATCATGAAGGGTGTGTGT	No Indel
MEK1 sgRNA2 OT4	chr7:67719250-67719272	-	4	TAAAATTA[AACATCACGGCT]	GGG	RP4-736H5.3	GCCTGAGCCTCCCGAATAGCTGG<br>GATGAGGCCCAGCTAGGTTACAG	No Indel
MEK1 sgRNA2 OT5	chr7:52788178-52788200	-	4	AAGAATGA[AACAGGACGGCT]	GGG	N/A	CGTAACTGGGACTACAGGCGTGTACC<br>CTGGACCCCCAACCCAATACACAA	No Indel
MEK1 sgRNA2 OT6	chr2:74963783-74963805	-	4	AAAATATA[ACAAGCACGGCT]	GGG	POLE4	CACTCATCCTTCAGCCTCTCAGGG<br>GGGAGTGCTGGGAGAAGGGAAATGA	No Indel
MEK1 sgRNA2 OT7	chr15:26017137-26017159	+	4	AAAACACA[AGCAACACGGCT]	GGG	RP11-1084I9.1	GCCCAGGTGGTTTACAAGGACAT<br>CTGAATGTGTTCCGGTGAGTGGGC	No Indel
MEK1 sgRNA2 OT8	chr5:157400207-157400229	+	4	CAAAAACA[AACAGCAAGGCT]	TGG	ADAM19	CCTGTATGCACAGGAAAAGGCACAAGC<br>CTTCCTTTAAATCAGCTATACTTCCCTGC	No Indel
MEK1 sgRNA2 OT9	chr4:155183086-155183108	-	4	AAAACTAG[AACAGCTCGGCT]	GGG	AC097467.2	CTCACTGCAAACTCCGTCTCCTGG<br>GTTTAGCAAAGTAGGCACCGCACAG	No Indel
MEK1 sgRNA2 OT10	chr17:2524519-2524541	-	4	AACATGCA[ATCAGCTCGGCT]	GGG	METTL16	GGTTTCAGTGAGCCGAGATT<br>AAAGACTCAGGAGAGCTCTAGG	No Indel

Supplementary Figure S6

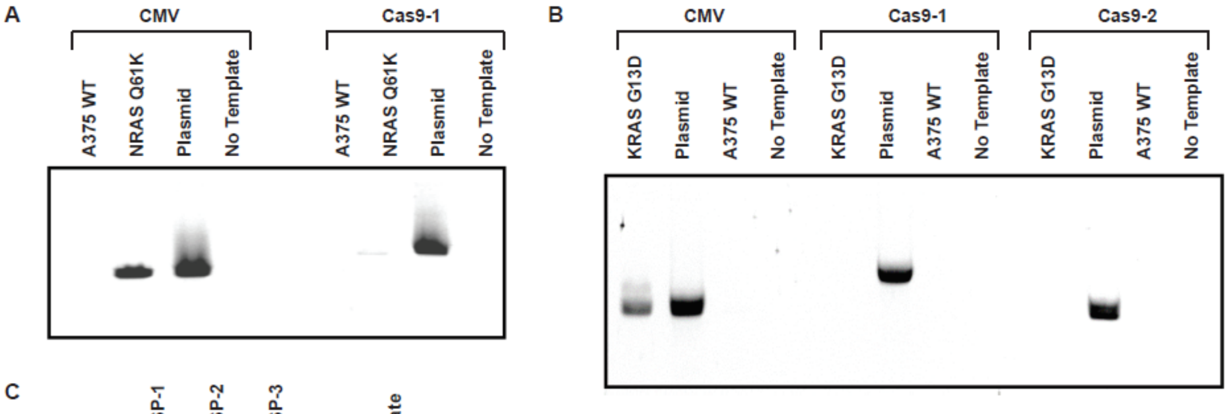

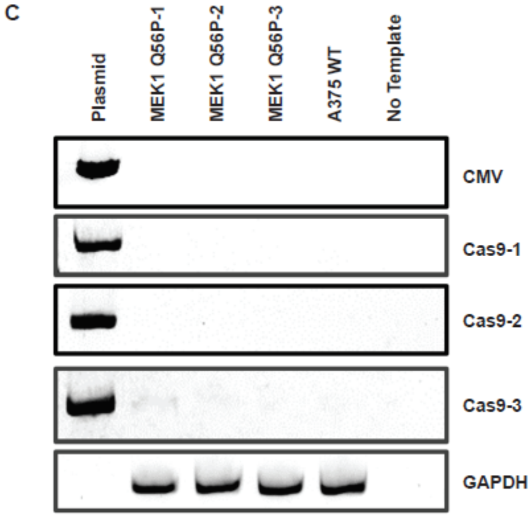

D

Primer	Sequence (5' to 3')	Amplicon
CMV-FW	GACATTGATTATTGACTAGTTATTAATAGTAA TCAATTACGGGGTCATTAGTTCATAGCC	583 bp
CMV-RV	AGCTCTGCTTATATAGACCTCCCACCGTACAC	
Cas9-1-FW	GCCACCATGGACAAGAAGTACTCCATTGG	999 bp
Cas9-1-RV	AGTCTTGGTGGTGCTCATCATAGCGC	
Cas9-2-FW	TTCAACTGAGCAAAGACACCTACGATGATG	556 bp
Cas9-2-RV	TACATAGTAGGGTATCCGAAATGTGAGG	
Cas9-3-FW	GGGACAGTCTTCACGAGCACATCG	1199 bp
Cas9-3-RV	CGCTGTTCCTTTTCGGGAGGATAC	
